# Evaluating the Diagnostic Value of Clinical and Laboratory Parameters in Older Adults with Abdominal Pain: A Retrospective Analysis of CT Predictors

**DOI:** 10.3390/medicina62071256

**Published:** 2026-06-29

**Authors:** Deniz Akyar, Nurseli Bayram, Ozge Ecmel Onur, Haldun Akoglu, Arzu Denizbasi

**Affiliations:** 1 Department of Emergency Medicine, Tokat Erbaa Public Hospital, 60500 Tokat, Turkey; denizakyar1979@gmail.com; 2 Department of Emergency Medicine, School of Medicine, Marmara University, 34899 Istanbul, Turkey; 3 Department of Emergency Medicine, Pendik Education and Research Hospital, Marmara University, 34899 Istanbul, Turkey

**Keywords:** abdominal pain, computed tomography, emergency department, geriatric patients, older adults

## Abstract

*Background and Objectives:* This study aims to evaluate easily accessible clinical and laboratory parameters in older adults presenting with nontraumatic abdominal pain and to explore factors associated with significant findings on abdominal computed tomography (CT). The goal is to help prevent diagnostic delays and reduce emergency department (ED) length of stay by minimizing unnecessary testing. *Materials and Methods:* This retrospective cohort study evaluated patients aged 65 years and older presenting to a high-volume tertiary ED with acute nontraumatic abdominal pain who underwent abdominal CT between January 2020 and January 2022. To maintain data integrity in a crowded ED environment, only patients with complete medical documentation were enrolled. Based on objective radiological outcomes from official reports, patients were categorized into two groups: those with Acute Pathological CT Findings (acute intra-abdominal pathology explaining the presentation) and those with Non-Acute/Negative CT Findings (normal scans, chronic, incidental, or extra-abdominal findings). Multivariable logistic regression was performed to identify independent predictors of acute pathological findings. *Results:* A total of 503 patients were included, of whom 178 (35.3%) had Acute Pathological CT Findings. Univariable analyses showed that elevated Alanine Aminotransferase (ALT), total bilirubin, and Gamma-Glutamyl Transferase (GGT) were significantly associated with acute pathological findings, whereas higher levels of Blood Urea Nitrogen (BUN), creatinine, and troponin were more prevalent in the Non-Acute/Negative CT Findings group. *Conclusions:* Despite the identified associations, a reliable predictive model could not be established; therefore, CT is considered to remain a fundamental tool for accurate diagnosis in older adults.

## 1. Introduction

With the increasing global population, the proportion of older adults is also rising, resulting in a growing number of older patients presenting to emergency departments (EDs). As in other age groups, abdominal pain is one of the most common complaints among older adult patients visiting ED [1,2]. However, due to age-related cognitive and physiological changes, establishing a diagnosis in this population can be particularly challenging for clinicians [3].

With advancing age, the weakening of the immune system—along with the presence of multiple comorbidities such as diabetes mellitus (DM), malignancy, cardiovascular disease (CVD), and dementia, as well as polypharmacy—often prevents older adult patients from mounting a full clinical response to underlying pathologies, unlike younger adults [2,3,4]. Moreover, because older adult patients may appear clinically stable on presentation and their laboratory values may initially be within normal limits, the diagnostic process can be delayed [3,5]. This increases the risk of misdiagnosis and may lead to premature discharge, which in turn can delay the initiation of appropriate treatment [3,5,6,7].

To address these diagnostic challenges in emergency settings, clinicians frequently attempt to utilize various clinical risk-stratification tools and decision-making algorithms. These range from specific acute abdomen protocols, such as the Alvarado or RIPASA scores for suspected appendicitis, to generalized geriatric vulnerability metrics, such as the Charlson Comorbidity Index or the Clinical Frailty Scale [8,9,10,11]. However, most traditional clinical and laboratory-based scoring systems were originally developed and validated within younger, healthier populations, often demonstrating poor diagnostic sensitivity and failed calibration when applied to the multi-layered physiological reality and blunt inflammatory pathways of older adults.

A comprehensive, validated prediction model structured strictly around easily accessible bedside presentation parameters for heterogenous geriatric abdominal pain cohorts remains a critical knowledge gap in the emergency medicine literature. Consequently, investigating whether routine clinical profiles can safely optimize or gatekeep advanced cross-sectional evaluation is crucial for modern geriatric care. In the crowded and chaotic environment of the ED, identifying reliable predictors that can guide emergency physicians toward appropriate advanced imaging is crucial for evaluating this vulnerable patient population.

Therefore, we aimed to investigate the predictive value of clinical and laboratory parameters in older adults presenting with acute abdominal pain. Through this study, we intend to provide clinicians with objective decision-making tools to prevent unnecessary advanced testing, thereby reducing prolonged ED lengths of stay that pose secondary risks to geriatric patients. Ultimately, minimizing unnecessary diagnostic workups will mitigate ED overcrowding and alleviate the associated economic burden on healthcare systems.

## 2. Materials and Methods

### 2.1. Study Design, Population, Inclusion and Exclusion Criteria

This retrospective cohort study was conducted at the Department of Emergency Medicine of Marmara University Pendik Training and Research Hospital, a high-volume, tertiary care academic medical center. Our ED serves as a major referral hub, accommodating a high-density patient influx with an average daily admission of approximately 1000 patients across green, yellow, and red triage categories. To ensure a comprehensive and unselected consecutive enrollment, data extraction was systematically performed via the institutional computerized hospital information system.

First, the electronic radiology department order logs were queried to retrieve all abdominal CT scans requested exclusively by the ED physicians for patients aged 65 years and older between 1 January 2020 and 1 January 2022. Following this initial electronic retrieval, individual electronic charts and official ED epicrises pre-existing in the hospital system were manually screened by the primary investigator for eligibility.

Patients were consecutively included in the final dataset if they were aged 65 years or older, presented with a clearly documented primary non-traumatic chief complaint of abdominal pain at ED presentation, and possessed a fully completed official epicrisis that explicitly detailed a confirmed final disposition diagnosis (discharge or admission outcome). Conversely, patients presenting with trauma-related abdominal symptoms, those transferred from external centers with pre-existing definitive diagnoses, and those who signed a refusal of treatment form were excluded.

Furthermore, due to the high-volume, fast-paced nature of our crowded tertiary ED environment, incomplete medical charting can occasionally occur during peak hours. To maintain strict data integrity and prevent subjective misinterpretation of clinical narratives, any patient whose electronic records lacked an explicit chief complaint, a verified final diagnosis, or the finalized primary outcome data in their official epicrisis was strictly excluded from the final analysis.

To ensure maximum data quality and eliminate investigator bias during data collection, the compiled electronic database was completely re-audited and cross-checked by a second independent emergency medicine researcher who was entirely blinded to the initial data extraction process. During this rigorous secondary validation phase, 34 patients with trauma-related abdominal pain and 18 patients with missing primary outcome data, who had been overlooked during the preliminary screening, were identified and strictly excluded from the study.

The study protocol was fully approved by the Clinical Research Ethics Committee of Marmara University Faculty of Medicine on 6 July 2022 (Protocol Code: 09.2022.826). Due to the retrospective nature of the study and the use of de-identified chart data, the requirement for informed consent was waived by the ethics committee.

### 2.2. Imaging Protocol and Adjudication

All abdominal CT examinations were performed in routine clinical practice using a 128-slice computed tomography scanner, utilizing a standardized reconstruction slice thickness of 5 mm. Both contrast-enhanced and non-contrast CT protocols were included in the final analysis based on the initial clinician ordering preference. The scans were interpreted and officially reported in routine clinical practice by board-certified attending radiologists via the institutional Picture Archiving and Communication System (PACS) as part of the standard clinical workflow.

In our institution’s routine practice, radiologists operate independently from the ED and perform interpretations based on the objective images, having access only to the brief clinical indication entered into the hospital system at the time of the CT order. For the purpose of this retrospective study, these pre-existing official radiology reports were utilized directly for data extraction, and no repeat image interpretations were performed by the research team.

To ensure complete objectivity and eliminate any investigator-driven selection bias, the research team did not make any subjective interpretations of the clinical or radiological data. Patients were classified into the primary outcome groups based strictly on the explicit, pre-existing final diagnoses documented in their official ED epicrises and consultation notes:

• Acute Pathological CT Findings (aCT): Restricted solely to patients whose official records contained an explicit, uncontradicted definitive diagnosis of an acute intra-abdominal emergency requiring acute intervention or admission (such as acute appendicitis, cholecystitis, bowel perforation, acute bowel obstruction, or acute intra-abdominal vascular events), as diagnosed by the treating clinical team in the routine workflow.
• Non-Acute/Negative CT Findings (nCT): Defined strictly as patients with completely normal scans, or those where the treating physicians and consultants explicitly documented chronic, benign, incidental, or extra-abdominal findings that were judged by the clinical team at the time of presentation to be entirely unrelated to an acute abdominal emergency.

No retrospective re-evaluation or diagnostic reconciliation was performed by the investigators; the documented clinical reality in the electronic medical records was accepted as the absolute ground truth.

### 2.3. Statistical Analysis

Statistical analyses were performed to evaluate the differences between the two primary study populations established based on radiological outcomes: the Acute Pathological CT Findings group (abbreviated as aCT) and the Non-Acute/Negative CT Findings group (abbreviated as nCT). All comparative and inferential analyses were strictly structured and conducted based on the variance between these two distinct groups.

Continuous variables were reported as means with standard deviations (SD) and 95% confidence interval (CI), or as medians with interquartile ranges (IQRs), depending on the distribution of the data. Categorical variables were reported as counts and percentages. Comparisons between outcome groups were made using the *t*-test or Mann–Whitney U test, based on data distribution. Categorical variables were compared using contingency tables and the chi-squared test. Univariable analysis of the predictor variables among outcome groups were also reported with odds ratios (ORs) and 95%CIs.

We also constructed a multivariable logistic regression model. Variables that were statistically significant in univariable analyses, along with clinically relevant variables, were entered into a multivariable logistic regression model. A type I error of 5% was accepted for all comparisons. All statistical analyses were performed using the R-based Jamovi statistical package (v2.6.44, 2025, Sydney, Australia).

## 3. Results

**Figure 1:**
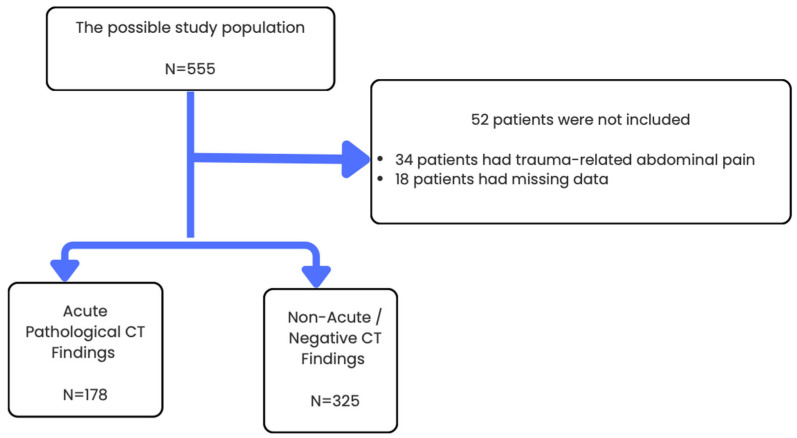
Flowchart of the study population enrollment process, demonstrating inclusion and exclusion criteria leading to the final analysis groups.

Between January 2020 and January 2022, a total of 555 patients aged 65 years and older who underwent abdominal CT imaging for non-traumatic abdominal pain in the ED were screened. After excluding 34 patients with trauma-related abdominal symptoms and 18 patients with missing primary outcome data, the final study cohort comprised 503 patients. Among these, 178 patients (35.3%) were classified into the Acute Pathological CT Findings group [YES (aCT)] and 325 patients (64.7%) were classified into the Non-Acute/Negative CT Findings group [NO (nCT)] (Figure 1).

In the overall study population, abdominal CT examinations revealed acute, non-acute, or incidental pathologic findings in 274 patients (54.5%). The most common radiological diagnoses were hepatobiliary disorders (13.7%), followed by genitourinary pathologies (9.7%), gastrointestinal disorders (8.2%), malignant masses (4.4%), and vascular emergencies (3.0%). Other heterogenous pathologies accounted for 12.9% of the findings, while 229 patients (45.5%) had completely normal abdominal CT scans.

The median age of the entire cohort was 75 years (IQR, 68–81). A statistically significant difference was observed in the median age between the aCT group (73 years; IQR, 67–79.8) and the nCT group (77 years; IQR, 69–82) (*p* < 0.001). Gender distribution was balanced, with 255 patients (50.7%) being female, and no statistically significant difference was found between the groups (*p* = 0.965).

Regarding overall clinical outcomes, 190 patients (37.8%) were safely discharged from the ED, 194 (38.6%) were admitted to general wards, 93 (18.3%) required intensive care unit (ICU) admission, and 13 patients (2.6%) died during their hospital stay. The baseline demographics, comorbidities, and physical examination findings stratified by the primary radiological outcome are comprehensively summarized in Table 1.

**Figure 2:**
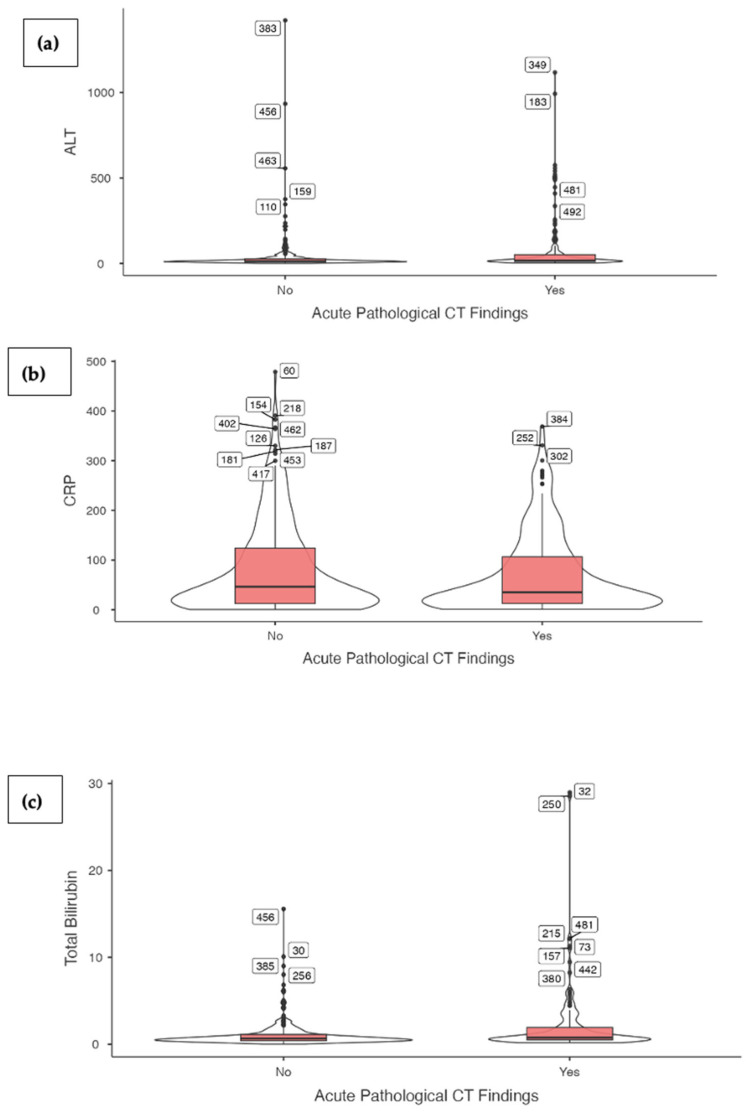
Combined violin and box plot representations of baseline laboratory parameters stratified by Acute Pathological CT Findings (N = 503): (**a**) Alanine Aminotransferase (ALT), (**b**) C-reactive Protein (CRP), and (**c**) Total Bilirubin. The heavily overlapping distribution bodies demonstrate that even when statistical significance is achieved (as in ALT and Bilirubin), the clinical magnitudes of differences remain narrow and concentrated within highly overlapping clinical ranges, while primary inflammatory markers like CRP remain entirely static at presentation.

Comparison of baseline laboratory parameters between the evaluation groups revealed statistically significant differences in several parameters, including hemoglobin (*p* = 0.002), red blood cell count (*p* = 0.001), ALT (*p* = 0.001), BUN (*p* < 0.001), creatinine (*p* < 0.001), troponin T (*p* < 0.001), total bilirubin (*p* = 0.004), direct bilirubin (*p* = 0.034), alkaline phosphatase (*p* = 0.005), GGT (*p* = 0.003), blood pH (*p* = 0.013), and base excess (*p* = 0.035). However, the absolute numerical magnitude of these laboratory differences remained narrow and within clinically non-specific, overlapping ranges. Most notably, standard emergency inflammatory biomarkers—including total white blood cell count (WBC) (*p* = 0.762), neutrophil percentage (*p* = 0.604), C-reactive protein (CRP) (*p* = 0.232), procalcitonin (*p* = 0.092), and lactate (*p* = 0.125)—demonstrated no statistical differences between the acute pathological and non-acute cohorts (Table 2 and Figure 2).

To identify independent predictors of acute pathological findings, variables demonstrating statistical significance in univariable screenings (*p* < 0.05), along with clinically forced demographic factors, were systematically evaluated using multivariable binomial logistic regression frameworks. Initially, an exhaustive multivariable model incorporating all 21 univariable significant laboratory and clinical parameters was constructed. However, due to severe data listwise deletion from non-simultaneous laboratory sampling in routine emergency flows, this comprehensive model was restricted to a limited subgroup (N = 166). Furthermore, this model demonstrated extensive statistical dissipation of laboratory parameters due to severe clinical collinearity, where prominent physical signs and patient baselines suppressed laboratory micro-variations. The full framework of this initial rigorous evaluation is comprehensively segregated and presented as Supplementary Table S1 to ensure full methodological transparency.

To optimize statistical power, eliminate missing-data selection bias, and construct a highly generalizable bedside screening framework, a refined multivariable binomial logistic regression analysis was executed. This final model focused strictly on core clinical and demographic baseline characteristics across the entire unselected sample cohort (N = 503; Model x^2^ = 78.369, df = 3, *p* < 0.001; Deviance = 575.34). The model demonstrated an excellent fit and accounted for a substantial proportion of variance, with a Nagelkerke R^2^ of 0.198.

In this refined clinical framework, the presence of physical abdominal tenderness was established as a robust, independent positive predictor of an acute intra-abdominal pathology, increasing the odds of aCT by more than three and a half times (Adjusted Odds Ratio [OR] = 3.55, 95% CI:2.39–5.27, *p* < 0.001). Conversely, advanced age (Adjusted OR = 0.97, 95% CI: 0.94–0.99, *p* = 0.013) and a care-dependent functional status (Adjusted OR = 0.22, 95% CI: 0.11–0.47, *p* < 0.001) maintained independent inverse statistical associations with the primary acute outcome group (Table 3). Variance Inflation Factor (VIF) screenings confirmed the total absence of collinearity among these predictors (VIF = 1.008).

To assess whether this clinical model could safely guide bedside decision-making and potentially gatekeep CT utilization, its predictive classification performance metrics were meticulously analyzed. Applying the standard clinical probability classification threshold of 0.5 yielded a moderate overall diagnostic accuracy of 70.6% and a solid specificity of 80.3%, correctly identifying 261 out of 325 patients within the negative nCT cohort.

**Figure 3:**
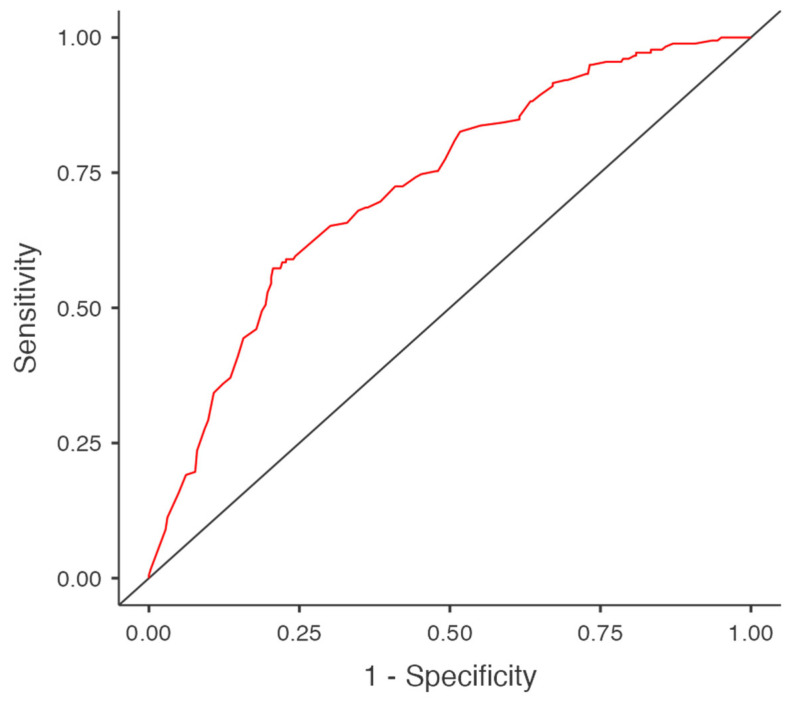
ROC Curve.

**Figure 4:**
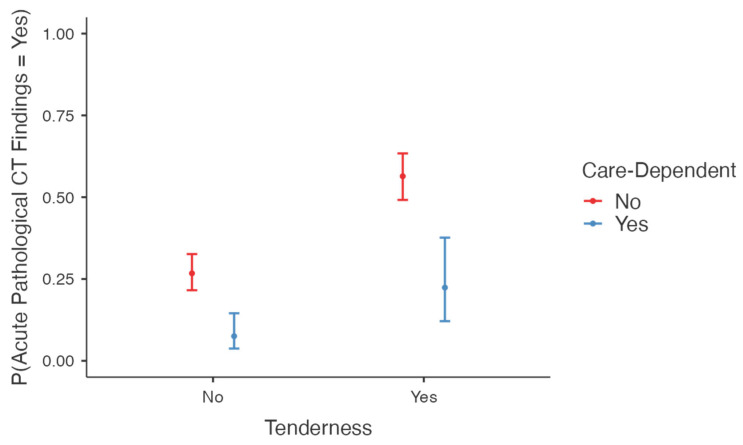
Estimated marginal means plot demonstrating the predicted probability of Acute Pathological CT Findings based on the interaction between physical abdominal tenderness and care-dependent status (N = 503). Vertical bars represent 95% confidence intervals. The standard classification cut-off probability threshold is set to 0.5.

However, the model exhibited an unacceptably low clinical sensitivity of 52.8%, misclassifying 84 out of 178 verified acute intra-abdominal crises (such as acute appendicitis, cholecystitis, or perforated viscus) as completely benign clinical profiles. Despite an acceptable cumulative mathematical discrimination (AUC= 0.724) (Figure 3), the substantial false-negative rate mathematically confirms that standalone or combined clinical presentation indices cannot safely replace cross-sectional abdominal CT imaging to rule out acute emergency pathology in older adult patients (Table 4 and Figure 4).

## 4. Discussion

In this study, which aimed to identify predictors of positive CT findings in older adults presenting with nontraumatic abdominal pain, hepatobiliary conditions were the most frequently identified diagnoses among patients with Acute Pathological CT findings, followed by gastrointestinal disorders. These results are consistent with previous reports in the literature [12,13,14,15]. The difficulty in identifying highly robust independent predictors and the moderate sensitivity of our model can be attributed to the profound physiological and clinical complexity inherent to the geriatric demographic. In older adults, the diagnostic utility of traditional indicators is frequently compromised by a high cumulative burden of multiple pre-existing comorbidities and atypical or entirely silent clinical presentations of acute diseases.

Furthermore, age-associated immunosenescence and the progressive blunting of classic neuro-inflammatory pathways can mask the typical systemic manifestations of acute intra-abdominal catastrophes. This physiological masking explains why standard emergency inflammatory biomarkers—including white blood cell count, neutrophil percentage, and C-reactive protein—demonstrated no statistical differences between our acute pathological and non-acute cohorts in the overall sample. With the exception of peripheral oxygen saturation (SpO_2_), no statistically significant differences were observed in vital signs between the aCT and nCT groups. Although SpO_2_ was independently associated with Acute Pathological CT findings in multivariable analysis, the small effect size suggests limited clinical relevance.

These findings are consistent with prior studies in older adults presenting to the ED with abdominal pain, which have reported similar admission vital parameters across diagnostic groups. Dadeh et al., Henden Cam et al., and Kumar et al. demonstrated that initial blood pressure, heart rate, and body temperature values were largely comparable among older patients with abdominal pain [15,16,17]. Collectively, these findings support existing evidence that older adults may present with relatively stable vital signs despite significant underlying pathology, potentially delaying diagnosis and underscoring the limited utility of vital parameters alone in guiding imaging decisions in this population [2,3]. This further supports the notion that vital signs alone may be insufficient to stratify risk in older adults with abdominal pain.

In this study, the median age of patients in the aCT group (73 years; IQR, 67–79) was significantly lower than that of the nCT group (77 years; IQR, 69–82). In addition, GCS scores ≥ 14 were significantly more frequent among patients with Acute Pathological CT findings (98.9%) compared with those without such findings (85.8%), and care dependency was significantly less common in the aCT group. Taken together, these associations may reflect that preserved cognitive status and functional independence allow for clearer symptom reporting, potentially aiding clinical decision-making rather than indicating a direct causal relationship.

Notably, our analysis revealed that advanced age and a care-dependent status maintained inverse statistical associations with acute pathological findings. Rather than indicating a true biological protection, this phenomenon likely reflects a well-documented selection and referral bias inherent to emergency triage patterns. Highly vulnerable or institutionalized older adults are frequently referred to the emergency department at much lower clinical thresholds—often for non-specific functional decline or mild change in status—leading to a broader utilization of defensive CT imaging within this sub-cohort. Because our study only included patients who underwent abdominal CT, this operational behavior mathematically lowers the proportion of acute pathologies discovered in these specific subgroups compared to independent older adults who typically present only after severe symptoms manifest.

This specific inverse association emphasizes a prominent operational selection and referral bias that must be factored into the generalizability of our findings. Because our enrollment protocol strictly captured patients for whom the attending ED physicians had already deemed cross-sectional imaging necessary, the study sample inherently reflects advanced clinician ordering behavior rather than an unselected baseline screening pool. Vulnerable, care-dependent, or ultra-elderly patients are frequently referred to tertiary emergency facilities with a lower clinical threshold for non-specific deterioration or minor status changes. Consequently, defensive medicine pathways prompt a wider, more conservative utilization of abdominal CT within these fragile subgroups, automatically driving down the proportion of positive acute findings relative to independent older adults who typically present only after severe, unmanageable symptoms manifest.

In this study, among the 178 patients in the aCT group, abdominal examination revealed tenderness in 109 patients (61.2%), guarding in 18 (10.1%), and rebound tenderness in 5 (2.8%). The relatively low frequency of guarding and rebound tenderness may be attributable to the subjective nature of these findings in older adult patients, as well as to age-related physiological changes that may blunt peritoneal signs. In addition, delayed or incomplete evolution of clinical signs at the time of ED presentation may further contribute to these observations. Importantly, the presence of abdominal tenderness alone on initial physical examination was associated with Acute Pathological CT findings in our cohort. This finding is consistent with the results reported by Southisombath et al., who demonstrated that abdominal tenderness was an independent predictor of abnormal abdominal CT findings in elderly patients presenting with acute abdominal pain [18]. Taken together, these findings suggest that while advanced peritoneal signs may be infrequent or unreliable in older adults, localized or diffuse abdominal tenderness may still represent a clinically meaningful indicator warranting further imaging evaluation.

Comorbid conditions were prevalent in the study population, affecting 460 patients (91.5%), including 154 patients in the aCT group (86.5%) and 306 in the nCT group (94.2%). Congestive heart failure (27.4% vs. 12.9%), cerebrovascular disease or transient ischemic attack (24.0% vs. 11.8%), coronary artery disease (45.8% vs. 29.2%), diabetes mellitus (48.3% vs. 35.4%), and care dependency (22.5% vs. 5.1%) were more common in the nCT group. In this older adult population, a high burden of comorbidity may be associated with increased diagnostic uncertainty and a lower threshold for imaging, rather than a direct association with Acute Pathological CT findings.

Several laboratory parameters differed statistically between patients with and without Acute Pathological CT findings. Patients in the aCT group had higher hemoglobin and red blood cell counts, as well as higher hepatobiliary markers, including alanine aminotransferase, alkaline phosphatase, gamma-glutamyl transferase, and total and direct bilirubin levels, whereas blood urea nitrogen, creatinine, and troponin T levels were higher in the nCT group. Although these differences reached statistical significance, their clinical relevance should be interpreted with caution. Importantly, the absence of significant differences in most inflammatory markers, including white blood cell count, C-reactive protein, procalcitonin, and lactate, suggests that laboratory parameters alone may have limited discriminatory value in this setting. Given the retrospective design and the limited statistical power of the study to detect small-to-moderate effect sizes, these findings should be considered exploratory rather than definitive. Larger, prospective studies are needed to clarify whether specific laboratory markers can reliably contribute to imaging decision-making in older adults presenting with abdominal pain.

This comprehensive statistical dissipation and the overall moderate performance of our predictive architecture are deeply rooted in the clinical and physiological complexity unique to the geriatric demographic. In older adult patients, a high cumulative burden of pre-existing chronic comorbidities, polypharmacy, and baseline functional decline drastically compromise the diagnostic precision of traditional clinical cues. More importantly, age-associated immunosenescence and the progressive systemic blunting of classic neuro-inflammatory pathways systematically mask or alter the expected metabolic and hematologic responses to acute intra-abdominal crises. This physiological masking directly accounts for the lack of clinical discrimination observed in standard inflammatory biomarkers—such as white blood cell count, neutrophil percentage, and C-reactive protein—which remained entirely static across our outcome cohorts. Consequently, our parameters mathematically demonstrate that conventional laboratory profiles lose their acute risk-stratification utility in older adults, necessitating advanced cross-sectional evaluation regardless of normal baseline blood windows.

Given the known limitations in history-taking and physical examination in the geriatric population, as well as the potential for misleading vital signs and laboratory results at presentation, computed tomography remains a crucial diagnostic tool for this specific patient group. In a study published by Esses et al. in 2004, which included 104 patients aged 65 and older who presented to the emergency department with nontraumatic abdominal pain, the authors compared the preliminary diagnoses made by clinicians based on history and physical examination with the final diagnoses established after abdominal CT imaging [19]. They found that CT results altered the initial diagnosis in 45% of cases (95% CI: 35–55) [19]. Additionally, the EDEN-43 study published by Miró et al. in 2025 aimed to identify potential risk factors associated with 30-day adverse outcomes—including mortality, emergency department revisits, and hospital admissions—in patients aged 65 years and older who were discharged from the emergency department with a diagnosis of nonspecific abdominal pain [20]. The study identified a high Charlson Comorbidity Index score, functional dependence, the need for analgesia, and the absence of laboratory and imaging investigations as independent risk factors for 30-day adverse events [20]. Consistent with the findings of the EDEN-43 study, strategic use of imaging is essential for mitigating risk in the geriatric population.

In our research, we sought to refine the diagnostic imaging process for older patients with abdominal pain, aiming to prevent unnecessary testing, decrease ED length of stay, and alleviate department overcrowding. When synthesized collectively, the 45% diagnostic alteration rate established by Esses et al. and the severe 30-day post-discharge adverse risks identified in the multicenter EDEN-43 framework provide a critical context for our model’s high false-negative threshold. Operating with an unacceptably low clinical sensitivity of 52.8%, our refined bedside criteria would theoretically misclassify nearly half of the verified acute emergencies as benign profiles if used as a clinical gatekeeper. The clinical application of these results validates the benchmarks of the EDEN-43 study; discharging or managing an older adult patient based strictly on stable presentation scores introduces an unsafe margin of error. Therefore, the mathematical reality of our model confirms that routine clinical and laboratory screening indices cannot safely substitute or ration computed tomography imaging without directly compromising geriatric patient safety.

## 5. Conclusions

In conclusion, our multivariable analysis demonstrates that we were unable to identify definitive or highly robust predictive parameters for acute intra-abdominal pathologies within this highly vulnerable demographic. The limited predictive power and the high false-negative rate observed in our classification model highlight the profound clinical and physiological complexity inherent to older adults. While our specific study design did not yield an ideal bedside risk-stratification score, we believe these findings carry considerable academic value by illustrating the challenges of relying solely on routine clinical or laboratory indices. Rather than providing definitive answers, our results outline a critical methodological reality and highlight the clear necessity for future, well-designed, prospective, multicenter trials with larger cohorts to develop comprehensive and clinically safe predictive scores for this patient group. Consequently, we anticipate that our current data, despite its lack of robust independent predictors, will serve as a foundational guide and a valuable reference for planning future clinical algorithms and stratifications in the geriatric emergency department setting.

### Limitations

Due to the retrospective nature of our study, the inability to access complete records limited the sample size. The lack of explicitly documented imaging indications for many patients who underwent abdominal CT led to their exclusion from the study. Evaluating only a single vital sign and a single laboratory parameter at presentation may not fully capture dynamic changes throughout the clinical course; furthermore, the time interval between pain onset and blood sampling was not standardized. Due to the retrospective design, we could not ensure that radiologists were blinded to clinical findings or preliminary diagnoses during reporting, which may introduce potential interpretation bias.

The poor diagnostic performance of our refined multivariable model and its inadequacy in guiding clinical decisions represent a major limitation; moreover, this weak model lacks external validation in an independent patient cohort. Because our database relied strictly on pre-existing official radiology reports rather than a standardized re-evaluation by a blinded research radiologist, the potential for inter-observer variability in radiological interpretations cannot be entirely excluded. Finally, our inclusion protocol was restricted exclusively to patients who underwent abdominal CT imaging. Consequently, rather than reflecting an unselected baseline screening pool of all geriatric abdominal pain presentations, our sample inherently reflects advanced clinician ordering behavior and defensive medicine pathways, further limiting the extrapolation of our weak predictive model.

## Figures and Tables

**Table 1:** Demographic Characteristics and Clinical Findings Stratified by Acute Pathological CT Findings.

Variable N (%)	Acute Pathological CT Findings		Difference in Proportions (95% CI)	*p*
	YES (aCT)	NO (nCT)		
Age, years; median (IQR), n = 503 (100.0)	73 (67–79.8); n = 178	77 (69–82); n = 325		<0.001 *
Female, n = 255 (100.0)	90 (50.6)	165 (50.8)	−0.002 (−0.085 to 0.082)	0.965 **
GCS ≥ 14, score, n = 458 (91.1)	176 (98.9)	282 (85.8)	−0.34 (−0.415 to −0.265)	<0.001 **
SBP, mmHg; median (IQR), n = 502 (100.0)	125 (110–146); n = 177	123 (106–145); n = 325		0.360 *
Pulse Rate, bpm; median (IQR), n = 493 (100.0)	92 (80–101); n = 173	90 (77–103); n = 320		0.578 *
Temperature, °C; median (IQR), n = 488 (100.0)	36.5 (36.1–36.7); n = 175	36.5 (36.2–36.8); n = 313		0.100 *
SpO_2_, %, median (IQR), n = 500 (100.0)	97 (95–98); n = 177	96 (91–98); n = 323		<0.001 *
History				
Comorbidity, n = 460 (91.5)	154 (86.5)	306 (94.2)	0.223 (0.069 to 0.378)	0.003 **
CHF, n = 112 (22.3)	23 (12.9)	89 (27.4)	−0.191 (−0.280 to −0.102)	<0.001 **
HT, n = 404 (80.3)	136 (76.4)	268 (82.5)	−0.088 (−0.195 to 0.020)	0.102 **
TIA/CVA, n = 99 (19.7)	22 (11.8)	78 (24.0)	−0.176 (−0.270 to −0.083)	<0.001 **
CAD, n = 201 (40.0)	52 (29.2)	149 (45.8)	−0.159 (−0.242 to −0.076)	<0.001 **
DM, n = 220 (43.7)	63 (35.4)	158 (48.3)	−0.120 (−0.203 to −0.037)	0.005 **
Care-dependent, n = 82 (16.3)	9 (5.1)	73 (22.5)	−0.292 (−0.374 to −0.209)	<0.001 **
Physical Examination				
Tenderness, n = 205 (40.8)	109 (61.2)	96 (29.5)	0.300 (0.217 to 0.384)	<0.001 **
Rebound tenderness, n = 5 (1.0)	5 (2.8)	0 (0.0)	0.653 (0.611 to 0.694)	0.005 ***
Guarding, n = 22 (4.4)	18 (10.1)	4 (1.2)	0.486 (0.319 to 0.652)	<0.001 **
CT Findings				
No pathology, n = 229 (45.5)	0 (0.0)	229 (70.5)		<0.001 **
Hepatobiliary ^1^, n = 69 (13.7)	40 (22.5)	29 (8.9)		
Appendicitis-diverticulitis, n = 2 (0.4)	2 (1.1)	0 (0.0)		
Pancreatitis, n = 11 (2.2)	11 (6.2)	0 (0.0)		
GUS ^2^, n = 49 (9.7)	29 (16.3)	20 (6.2)		
GIS ^3^, n = 41 (8.2)	39 (21.9)	2 (0.6)		
Vascular pathology ^4^, n = 15 (3.0)	10 (5.6)	5 (1.5)		
Malignancy, n = 22 (4.4)	18 (10.1)	4 (1.2)		
Other, n = 65 (12.9)	29 (16.3)	36 (11.1)		

* Mann–Whitney U ** Chi-square *** Fisher’s exact test. IQR: Interquartile range; CT: computed tomography; GCS: Glasgow Coma Scale; SBP: systolic blood pressure; SpO_2_: peripheral oxygen saturation; CHF: congestive heart failure; HT: hypertension; TIA: transient ischemic attack; CVA: cerebrovascular accident; CAD: coronary artery disease; DM: diabetes mellitus; GUS: genitourinary system; GIS: gastrointestinal system; ^1^: cholecystitis, cholelithiasis, cholangitis, hepatitis, abscess; ^2^: nephro/urolithiasis, pyelonephritis, abscess; ^3^: perforation, ileus, colitis, ileitis; ^4^: mesenteric ischemia, infarction, aortic dissection, hemorrhage.

**Table 2:** Relationship Between Baseline Laboratory Parameters and Acute Pathological CT findings.

Variable	N (%) N = 503	Acute Pathological CT Findings				*p* *
		YES (aCT)		NO (nCT)		
		N = 178	Median (IQR)	N = 325	Median (IQR)	
WBC, ×10^3^/uL			9.9 (7.4 to 13.1)		9.8 (6.8 to 18.8)	0.762
Lymphocyte, %			11.1(6.7 to 18.8)		11.5 (6.5 to 19.4)	0.753
Neutrophil, %			80.1(71.0 to 86.4)		79 (69.4 to 86.6)	0.604
Hgb, g/dL			12.2 (10.4 to 13.1)		11.1 (9.4 to 13.0)	0.002
RBC, ×10^6^/uL			4.2 (3.6 to 4.7)		3.9 (3.4 to 4.5)	0.001
ALT, IU/L			17.1 (12.0 to 51.7)		14.7 (10.0 to 27.9)	0.001
AST, IU/L			26.9 (19.0 to 27.9)		25 (17.9 to 43.0)	0.093
BUN, mg/dL			22 (16.2 to 30.7)		32 (20.0 to 58.0)	<0.001
Creatinine, mg/dL	501		1 (0.75 to 1.48)	323	1.4 (0.9 to 2.4)	<0.001
CRP, mg/L	486	171	35.4 (12.5 to 106.9)	315	46.5 (12.4 to 124.1)	0.232
PCT, ug/L	433	159	0.21 (0.08 to 1.14)	274	0.31(0.1 to 1.3)	0.092
D-Dimer, mg/L	95	15	2.36 (1.08 to 6.55)	80	2.36 (1.08 to 6.55)	0.939
T. Bilirubin, mg/dL	428	158	0.76 (0.49 to 1.94)	270	0.66 (0.43 to 1.12)	0.004
D. Bilirubin, mg/dL	429	158	0.25 (0.14 to 0.78)	271	0.21 (0.13 to 0.38)	0.034
Amylase, IU/L	415	156	53.5 (35.7 to 83.5)	259	57 (35.0 to 83.0)	0.951
Lipase, IU/L	419	156	25 (15.0 to 47.4)	263	25 (15.0 to 43.9)	0.938
Troponin T, ng/L	196	41	19.3 (13.2 to 36.2)	155	39.2 (21.2 to 89.3)	<0.001
LDH, IU/L	416	151	275 (234.0 to 387.0)	265	305 (235.0 to 387.0)	0.176
ALP, IU/L	426	156	105.5 (78.7 to 189.5)	270	94 (71.0 to 143.7)	0.005
GGT, IU/L	420	156	42.9 (17.9 to 178.0)	264	29.0 (17.2 to 62.5)	0.003
pH	493	176	7.38 (7.35 to 7.41)	317	7.37 (7.32 to 7.41)	0.013
HCO^3^, mmol/L	493	176	24.4 (21.3 to 26.8)	317	23.8 (19.9 to 26.5)	0.088
BE, mmol/L	493	176	0.05 (−3.15 to 1.82)	317	−0.9 (−4.9 to 1.8)	0.035
Lactate, mmol/L	493	176	2.45 (1.7 to 3.2)	317	2.5 (1.9 to 3.5)	0.125

* Mann–Whitney U IQR: Interquartile range; CT: computed tomography; WBC: White Blood Cell; RBC: Red Blood Cell; ALT: Alanine Aminotransferase; AST: Aspartate Aminotransferase; BUN: Blood Urea Nitrogen; CRP: C-Reactive Protein; PCT: Procalcitonin; LDH: Lactate Dehydrogenase; ALP: Alkaline Phosphatase; GGT: Gamma-Glutamyl Transferase.

**Table 3:** Refined Multivariable Logistic Regression Analysis of Clinical Factors Associated with Acute Pathological CT Findings (N = 503).

						95% Confidence Interval	
Predictor	Estimate	SE	Z	*p*	Odds Ratio	Lower	Upper
Intercept	1.438	0.981	1.466	0.143	4.213	0.616	28.832
Age	−0.032	0.013	−2.484	0.013	0.968	0.944	0.993
Care-Dependent:							
Yes–No	−1.501	0.379	−3.965	<0.001	0.223	0.106	0.468
Tenderness:							
Yes–No	1.266	0.202	6.268	<0.001	3.547	2.387	5.269

Note. Estimates represent the log odds of “Acute Pathological CT Findings = Yes” vs. “Acute Pathological CT Findings = No”. Overall Model Test: x^2^ = 78.369, df = 3, *p* < 0.001; Nagelkerke R^2^ = 0.198.

**Table 4:** Classification Matrix and Diagnostic Performance Metrics of the Refined Clinical Model (N = 503).

Performance Metric Indicator	Statistical Value	Observed Classification Matrix	Predicted Negative (nCT)	Predicted Positive (aCT)	Percentage Correct
Overall Accuracy	0.706	Observed Negative (nCT)	261	64	80.3%
Sensitivity	0.528	Observed Positive (aCT)	84	94	52.8%
Specificity	0.803				
Area Under ROC (AUC)	0.724	Overall Sample Accuracy			70.6%

## Data Availability

The datasets used and/or analysed during the current study are available from the corresponding author on reasonable request.

## Supplementary Materials

The following supporting information can be downloaded at: https://www.mdpi.com/article/10.3390/medicina62071256/s1. Table S1. Comprehensive multivariable logistic regression parameters, omnibus likelihood ratio tests, and model fit measures for clinical and laboratory predictors of acute pathological CT findings (N = 166); Figure S1: ROC curve for the comprehensive multivariable model (N = 166).

## Abbreviations

The following abbreviations are used in this manuscript:

aCT	Acute Pathological CT Findings
ALT	Alanine Aminotransferase
BUN	Blood Urea Nitrogen
CAD	coronary artery disease
CHF	congestive heart failure
CI	confidence interval
CT	computed tomography
CVA	cerebrovascular accident
CVD	cardiovascular disease
DM	diabetes mellitus
ED	emergency department
GCS	Glasgow Coma Scale
GGT	Gamma-Glutamyl Transferase
GIS	gastrointestinal system
GUS	genitourinary system
HT	hypertension
ICU	Intensive Care Unit
IQR	interquartile ranges
nCT	Non-Acute/Negative CT Findings
OR	odds ratio
SBP	systolic blood pressure
SD	standard deviation
SpO_2_	peripheral oxygen saturation
TIA	transient ischemic attack.
