# Supplementary material for: Evaluating the Diagnostic Value of Clinical and Laboratory Parameters in Older Adults with Abdominal Pain: A Retrospective Analysis of CT Predictors
Source: Medicina (Kaunas). 2026 Jun 29;62(7):1256. doi: 10.3390/medicina62071256 (PMC13413764; doi:10.3390/medicina62071256)

Supplementary Table S1. Comprehensive multivariable logistic regression parameters, omnibus likelihood ratio tests, and model fit measures for clinical and laboratory predictors of acute pathological CT findings (N=166).

Model Fit Measures

					Overall Model Test		
Model	Deviance	AIC	BIC	R^2_N	χ^2	df	p
1	121.742	165.742	234.206	0.452	59.257	21	<.001

Note. Models estimated using sample size of N=166.

Omnibus Likelihood Ratio Tests

Predictor	χ^2	df	p
Age	2.024	1	0.155
Tenderness	4.474	1	0.034
Care-Dependent	5.288	1	0.021
SpO_2	3.838	1	0.050
DM	0.238	1	0.626
CHF	0.028	1	0.868
History of TIA/CVA	0.000	1	1.000
CAD	5.890	1	0.015
GCS-	7.506	1	0.006
RBC	0.980	1	0.322
HGB	0.970	1	0.325
ALT	4.832	1	0.028
BUN	0.223	1	0.637
Creatinine	1.757	1	0.185
Total Bilirubin	0.039	1	0.843
Direct Bilirubin	0.143	1	0.706
Troponin T	0.380	1	0.538
ALP	0.097	1	0.756
GGT	2.324	1	0.127
pH	0.078	1	0.780
BE	0.507	1	0.476

Model Coefficients - Acute Pathological CT Findings

Predictor	Estimate	SE	Z	p	Odds Ratio	95% Confidence Interval	
						Lower	Upper
Intercept	-17.283	34.936	-0.495	0.621	0.000	0.000	1.7041102897743323e+22
Age	-0.049	0.035	-1.393	0.163	0.952	0.889	1.020
Tenderness:							
Yes – No	1.050	0.504	2.085	0.037	2.859	1.065	7.673
Care-Dependent:							
Yes – No	-1.863	0.904	-2.060	0.039	0.155	0.026	0.913
SpO_2	0.116	0.064	1.811	0.070	1.123	0.991	1.272
DM:							
Yes – No	-0.265	0.545	-0.486	0.627	0.767	0.264	2.231
CHF:							
Yes – No	0.100	0.600	0.166	0.868	1.105	0.341	3.585
History of TIA/CVA:							
Yes – No	0.000	0.594	0.000	1.000	1.000	0.312	3.206
CAD:							
Yes – No	-1.401	0.599	-2.339	0.019	0.246	0.076	0.797
GCS-:							
0 – 1	-24.505	1422.370	-0.017	0.986	0.000	0.000	Inf
RBC	0.557	0.562	0.991	0.322	1.745	0.580	5.252
HGB	-0.188	0.191	-0.987	0.324	0.828	0.570	1.204
ALT	0.010	0.005	1.789	0.074	1.010	0.999	1.020
BUN	0.008	0.017	0.478	0.633	1.008	0.976	1.042
Creatinine	-0.567	0.452	-1.256	0.209	0.567	0.234	1.375
Total Bilirubin	-0.117	0.589	-0.199	0.843	0.890	0.281	2.820
Direct Bilirubin	-0.327	0.882	-0.371	0.711	0.721	0.128	4.062
Troponin T	-0.001	0.002	-0.576	0.565	0.999	0.995	1.003
ALP	-0.001	0.005	-0.310	0.757	0.999	0.990	1.007
GGT	0.004	0.003	1.391	0.164	1.004	0.998	1.011
pH	1.307	4.675	0.280	0.780	3.695	0.000	35221.234
BE	-0.052	0.074	-0.709	0.478	0.949	0.821	1.097

Note. Estimates represent the log odds of "Acute Pathological CT Findings = Yes" vs. "Acute Pathological CT Findings = No".

Assumption Checks

Collinearity Statistics

	VIF	Tolerance
**Age**	1.421	0.704
**Tenderness**	1.237	0.809
**Care-Dependent**	1.232	0.812
**SpO_2**	1.209	0.827
**DM**	1.395	0.717
**CHF**	1.384	0.723
**History of TIA/CVA**	1.178	0.849
**CAD**	1.667	0.600
**GCS-**	1.000	1.000
**RBC**	5.384	0.186
**HGB**	5.415	0.185
**ALT**	1.752	0.571
**BUN**	3.461	0.289
**Creatinine**	4.114	0.243
**Total Bilirubin**	12.490	0.080
**Direct Bilirubin**	12.517	0.080
**Troponin T**	1.184	0.844
**ALP**	3.439	0.291
**GGT**	2.691	0.372
**pH**	2.989	0.335
**BE**	3.177	0.315

Prediction

Classification Table

	Predicted		
**Observed**	**No**	**Yes**	**% Correct**
No	122	5	96.063
Yes	22	17	43.590

Note. The cut-off value is set to 0.5.

Predictive Measures

Accuracy	Specificity	Sensitivity	AUC
0.837	0.961	0.436	0.866

Note. The cut-off value is set to 0.5.

Figure S1: ROC curve for the comprehensive multivariable model (N = 166).

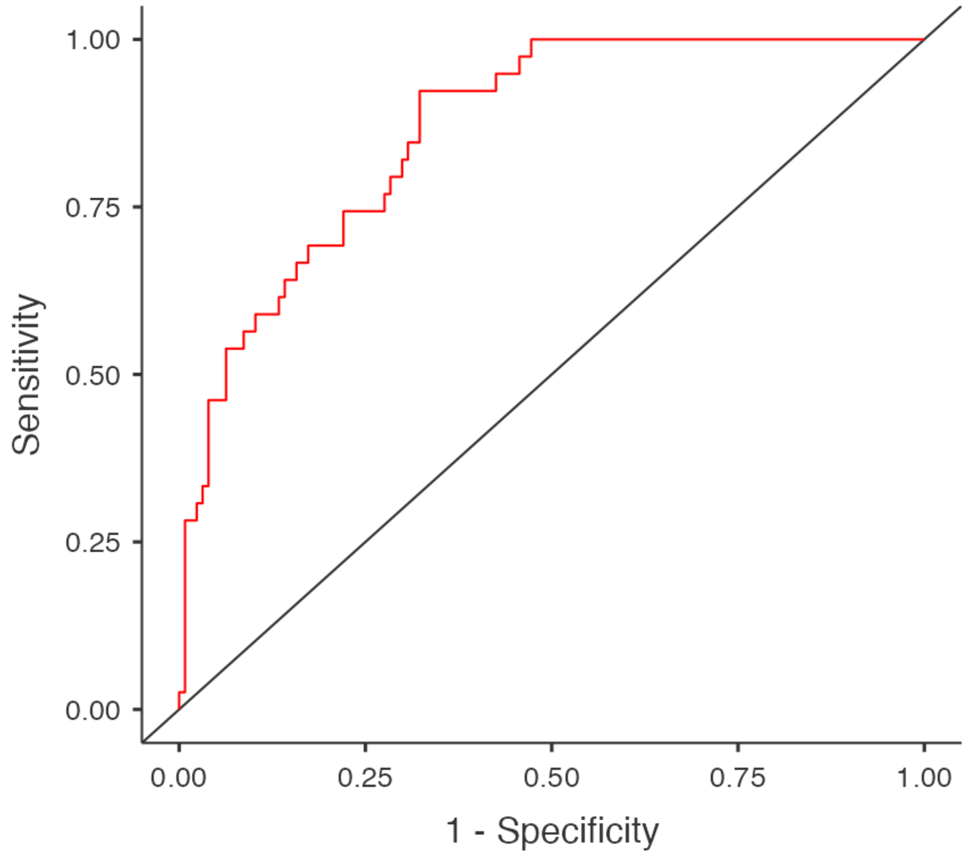

Supplement: Supplementary file 1 [file medicina-62-01256-s001.zip › medicina-4332555-supplementary.pdf]
